# Diagnostic Puzzles and Cause-Targeted Treatment Strategies in Myocardial Infarction with Non-Obstructive Coronary Arteries: An Updated Review

**DOI:** 10.3390/jcm12196198

**Published:** 2023-09-26

**Authors:** Athanasios Samaras, Dimitrios V. Moysidis, Andreas S. Papazoglou, Georgios Rampidis, Polydoros N. Kampaktsis, Konstantinos Kouskouras, Georgios Efthymiadis, Antonios Ziakas, Nikolaos Fragakis, Vasileios Vassilikos, George Giannakoulas

**Affiliations:** 1 First Department of Cardiology, AHEPA University Hospital, School of Medicine, Faculty of Health Sciences, Aristotle University of Thessaloniki, 546 36 Thessaloniki, Greece; ath.samaras.as@gmail.com (A.S.); dimoysidis@gmail.com (D.V.M.); aspapazo@auth.gr (A.S.P.); grampidi@gmail.com (G.R.); geythymi@auth.gr (G.E.); aziakas@auth.gr (A.Z.); 2 Second Cardiology Department, Hippokration General Hospital of Thessaloniki, 546 42 Thessaloniki, Greece; nfrag@auth.gr; 3 Third Cardiology Department, Hippokration General Hospital of Thessaloniki, 546 42 Thessaloniki, Greece; vvassil@auth.gr; 4 Department of Medicine, Columbia University Irving Medical Center/NewYork-Presbyterian Hospital, New York, NY 10032, USA; pkampaktsis@yahoo.com; 5 Department of Radiology, AHEPA University General Hospital of Thessaloniki, School of Medicine, Faculty of Health Sciences, Aristotle University of Thessaloniki, 541 24 Thessaloniki, Greece; coskou@auth.gr

**Keywords:** myocardial infarction with non-obstructive coronary arteries (MINOCA), pathophysiology and diagnosis, cause-targeted therapy

## Abstract

Myocardial infarction with nonobstructive coronary arteries (MINOCA) is a distinct subtype of myocardial infarction (MI), occurring in about 8–10% of spontaneous MI cases referred for coronary angiography. Unlike MI with obstructive coronary artery disease, MINOCA’s pathogenesis is more intricate and heterogeneous, involving mechanisms such as coronary thromboembolism, coronary vasospasm, microvascular dysfunction, dissection, or plaque rupture. Diagnosing MINOCA presents challenges and includes invasive and non-invasive strategies aiming to differentiate it from alternative diagnoses and confirm the criteria of elevated cardiac biomarkers, non-obstructive coronary arteries, and the absence of alternate explanations for the acute presentation. Tailored management strategies for MINOCA hinge on identifying the underlying cause of the infarction, necessitating systematic diagnostic approaches. Furthermore, determining the optimal post-MINOCA medication regimen remains uncertain. This review aims to comprehensively address the current state of knowledge, encompassing diagnostic and therapeutic approaches, in the context of MINOCA while also highlighting the evolving landscape and future directions for advancing our understanding and management of this intricate myocardial infarction subtype.

## 1. Introduction

Acute myocardial infarction, a critical manifestation of coronary artery disease (CAD), is typically associated with significant coronary artery stenosis or occlusion [1]. However, myocardial infarction with non-obstructive coronary arteries (MINOCA) has emerged during the last two decades as a distinct clinical entity in the absence of obstructive coronary artery with stenosis ≥ 50% in any potential infarct-related artery disease, posing unique diagnostic and therapeutic challenges [2,3]. MINOCA has been shown to represent approximately 10% of acute coronary syndromes [4,5,6,7]. This enigmatic clinical condition shares heterogeneous pathogenesis, which leads to poorly understood underlying mechanisms and suboptimal therapeutic and prevention strategies.

Recent studies have indicated several potential causes of MINOCA, highlighting coronary microvascular dysfunction, plaque erosion, coronary artery vasospasm, and coronary embolism as the most common [8,9,10,11]. Understanding the underlying mechanisms contributing to MINOCA is crucial for the development of cause-targeted, effective therapeutic approaches [3]. The current management of MINOCA patients typically involves the administration of standard therapy for myocardial infarction with obstructive CAD, such as antiplatelet agents, beta-blockers, and angiotensin-converting enzyme (ACE) inhibitors [10,12,13,14]. However, evidence for the effectiveness of these therapies in MINOCA remains limited, and their application is often based on extrapolation from studies in obstructive CAD patients [15]. Novel therapeutic approaches targeting specific underlying mechanisms of MINOCA, such as endothelial dysfunction or inflammation, have shown promising results in preclinical studies [16,17]. A critical evaluation of the preclinical and clinical evidence of these cause-targeted therapies could provide valuable insights into their potential role in the management of MINOCA patients.

This review aims to provide a comprehensive and systematic analysis of the existing literature on MINOCA, encompassing clinical trials, observational studies, and mechanistic research, to elucidate the pathophysiological basis of MINOCA and evaluate the potential efficacy of cause-targeted therapies. We also intend not only to address but also to fill the existing gaps in the understanding of MINOCA pathophysiology and treatment options, serving as a valuable resource for clinicians, researchers, and healthcare policymakers. By exploring cause-targeted therapies and their potential benefits, future personalized and evidence-based treatment strategies could be achieved, ultimately improving the quality of care, outcomes, and quality of life for patients suffering from this complex and challenging condition [18].

## 2. Pathophysiologic Mechanisms

The pathogenesis of MINOCA is heterogeneous and encompasses atherosclerotic plaque rupture or erosion, coronary thromboembolism, coronary vasospasm, coronary microvascular dysfunction, coronary dissection, etc. [3]. In some cases, patients may have an initial diagnosis of MINOCA, but additional testing reveals a diagnosis of acute myocarditis or Takotsubo syndrome. These patients are not considered to have MINOCA once a non-ischemic diagnosis has been established [3]. The lack of proper understanding of the mechanisms of MINOCA could potentially lead to suboptimal secondary prevention measures [19]. Therefore, both ESC guidelines and the AHA Scientific Statement highlight the role of cause-targeted therapies in MINOCA, suggesting that these patients should be treated according to the underlying pathophysiological mechanism [3,20,21]. Of note, in the recent 2023 ESC acute coronary syndromes (ACS) guidelines, the management of MINOCA according to the final established underlying diagnosis is highly recommended, while further diagnostic testing to determine the underlying final diagnosis is deemed of paramount importance (Class IB) [21].

### 2.1. Plaque Disruption

Coronary plaque disruption is a common cause of MINOCA, mainly including plaque rupture and plaque erosion; Specifically, its prevalence was 38% in an IVUS study [22] and 16–40% in OCT/CMR studies [23,24,25,26]. Patients with MINOCA experience a similar pathophysiology to those with MI and obstructive CAD, except that the obstruction should not surpass 50% [27]. Culprit atherosclerotic plaques in patients with MINOCA are typically smaller compared to patients with obstructive MI-CAD and are rarely outwardly remodeled [9,23]. When plaque rupture occurs, tissue factor and thrombogenic contents from the lipid-rich necrotic core are exposed to the bloodstream, leading to local thrombus formation [28]. Another possible atherosclerotic mechanism in MINOCA is plaque erosion, which involves thrombus formation over a denuded surface endothelium, which in turn overlays a fibrous plaque rather than a lipidic plaque [29].

MINOCA can result from various mechanisms associated with plaque rupture and erosion:

- Transient vessel occlusion may occur at the rupture site before intrinsic thrombolysis. Spontaneous thrombolysis or autolysis of a coronary thrombosis has been proposed as an explanation for the absence of sub-occlusive or occlusive thrombi during coronary angiography in MINOCA, especially when the procedure is performed late, and antithrombotic and antiplatelet agents are promptly administered [9];
- Distal embolization of the thrombus, with or without angiographically evident small-vessel occlusion, is another possibility;
- Flush occlusion of the ostium of a side branch may also occur without apparent signs in invasive angiography.

In patients with MINOCA without subcritical plaque disruption, CMR imaging might reveal large areas of myocardial edema with or without small areas of necrosis, indicating transient impairment of flow in a larger vessel. Alternatively, CMR may show a smaller, well-defined LGE area associated with a smaller vessel, suggesting the embolization of atherothrombotic debris from the rupture site as the most likely mechanism of myonecrosis [22]. Angiographic features that could suggest the occurrence of plaque rupture include mild vessel narrowing (<50%), asymmetrical lesions, narrow neck, irregular edges, haze, or a radio-lucent flap [3,30].

### 2.2. Epicardial Coronary Vasospasm 

Coronary artery spasm (CAS) is a common mechanism of MINOCA. It occurs due to vascular smooth muscle hyperreactivity to endogenous or exogenous vasospastic substances, leading to myocardial ischemia and infarction with electrocardiographic changes in the absence of coronary artery obstruction [31]. The clinical presentation of CAS is most frequently related to variant angina. In contrast to classical exercise-induced angina pectoris, variant angina occurs at rest, frequently during sleep or early in the morning, independent of myocardial oxygen demand, although exercise can still trigger chest pain and CAS in a significant number of patients. In variant angina, CAS occurs in normal or nearly normal epicardial vessels in approximately 50% of patients. Consequently, in these cases, MINOCA may occur when a prolonged episode of CAS leads to ischemic myocardial injury [32].

The provocative coronary spasm test with intracoronary acetylcholine or ergonovine infusion with epicardial vasospasm visualization on coronary angiography is considered the gold standard for diagnosis of coronary epicardial vasospasm [33,34,35]. This test was positive in a relatively large proportion of patients with MINOCA, suggesting that epicardial coronary vasospasm is a common pathogenetic mechanism in MINOCA [9,36].

Epicardial and microvascular vasospasms are attributed to endothelial dysfunction at the arterial and arteriolar levels, respectively [37]. Epicardial spasm is characterized by vasoconstriction of at least 90% following provocation testing [31]. Microvascular spasm is characterized by the manifestation of symptoms along with changes in the electrocardiogram (ECG) in response to a provocation without any epicardial spasm [38]. Among a series of 80 consecutive MINOCA patients, 37 individuals (46.2%) exhibited evidence of coronary spasm, with 24 of them diagnosed with epicardial spasm and 13 with microvascular spasm [9]. The presence of any form of coronary spasm was associated with a higher risk of long-term mortality (32.4% versus 4.7%, *p* = 0.002), and epicardial spasm was linked to more unfavorable outcomes compared to microvascular spasm [9]. In a separate group of 40 patients without intravascular imaging evidence of a high-risk coronary culprit lesion to explain the MINOCA presentation, 38% had a positive provocation test for coronary spasm [26]. Ong et al. reported in the CASPAR study that as many as 50% of patients with MINOCA experienced coronary vasospasm following acetylcholine infusion [35], and multiple studies indicate that this test is well-tolerated in the context of MI [9,34].

Both coronary spasm and atherosclerotic culprit lesions can coexist in the same patient and may even be present in the same coronary segment [23,39]. Considering the high prevalence of coronary spasm in MINOCA cases, it is reasonable to consider spasm as the potential cause of MINOCA in situations where infarction or regional edema is detected by cardiac magnetic resonance (CMR) imaging and no coronary culprit lesion is found during intracoronary imaging [23]. In such cases, the presence of spasm may be inferred as a plausible explanation for the MINOCA presentation without the need for dedicated provocative testing.

Numerous studies indicate that performing provocative testing during index hospitalization or by referring patients to capable facilities can provide several advantages. Firstly, a positive provocative test in MINOCA is considered safe, as the occurrence of arrhythmias is rare [33,35]. Secondly, provocative testing can be valuable in identifying MINOCA patients who may have poor outcomes [9,40]. Most importantly, establishing a diagnosis through provocative testing increases the likelihood of prescribing appropriate medical therapy, including CCB and other effective antianginal medications for vasospasm, upon discharge. This targeted approach ensures that patients receive the most suitable treatment to manage and prevent future episodes of vasospastic angina, leading to improved long-term outcomes and quality of life [9].

### 2.3. Coronary Microvascular Dysfunction

Coronary microvascular disease (CMD) impedes the necessary increase in coronary blood flow in response to increased cardiac oxygen requirements and can lead to myocardial ischemia [12]. Specifically, increased microvascular resistance, vasoreactivity, and impaired vasodilation can cause a supply–demand mismatch that results in hypoperfusion during hyperemic states. This condition is associated with the development of heart failure with preserved ejection fraction, diabetes mellitus, hypertension, and a series of unfavorable clinical outcomes [41]. Even though adequate data are lacking, it has been estimated that CMD accounts for almost 20% of patients with MINOCA [2,36]. 

CMD is diagnosed with a coronary flow reserve (CFR) < 2 after vasodilator administration, a value of an index of microvascular resistance (IMR) greater than 25, or a corrected TIMI frame count ≥ 3 beats to fill a vessel [38,42]. A reduced CFR signifies the vasculature’s inability to dilate and increase coronary blood flow adequately to meet the metabolic demands during hyperemic states [43]. The results of a positive intracoronary adenosine test do not provide a distinction between ischemic and nonischemic insults since CMD can occur in cases of nonischemic myocardial injury, such as myocarditis [4]. There are limited data regarding the utility of coronary microvascular testing in MINOCA. While CMD is definitively diagnosed through invasive coronary functional testing, alternative noninvasive methods can also be employed for diagnosis, including transthoracic Doppler, stress CMR, or positron emission tomography (PET) [38,41].

Functional CMD has been related to the presence of vasodilator abnormalities, as well as to an increased constriction of coronary microvessels (microvascular spasm). Endothelium-dependent or endothelium-independent mechanisms have been associated with the presence of impaired dilation [44,45]. CMD may also be the result of structural alterations, especially in patients with risk factors for coronary artery disease or with underlying cardiomyopathies. Luminal narrowing of the intramural arterioles and capillaries, perivascular fibrosis, and capillary rarefaction are the main mechanisms associated with structural abnormalities associated with CMD [46].

### 2.4. Spontaneous Coronary Artery Dissection

Spontaneous coronary artery dissection (SCAD) is an uncommon cause of MINOCA, with an estimated prevalence ranging from 1.7% to 4% [23]. SCAD is also the predominant cause of MI associated with pregnancy and occurs in the first six months postpartum (most often) or during pregnancy [47]. In SCAD, the formation of an intramural hematoma causes narrowing of the true lumen, blocking side branches, or both, ultimately resulting in an MI. Even though SCAD is not caused by atherosclerosis, it often appears as a significant coronary narrowing on coronary angiography, making it an angiographic diagnosis that is not congruent with MINOCA. Consequently, in some instances, SCAD may be misinterpreted as an MI related to atherosclerosis or thrombosis [48]. SCAD is a commonly overlooked cause of MINOCA that requires a high index of suspicion [3]. There are three types of SCAD categorized based on their angiographic appearance. Type 1 SCAD shows contrast stains in the arterial wall, presenting with multiple radiolucent lumens, either with or without slow contrast clearing [49,50]. Type 2 SCAD manifests as a diffuse and smooth narrowing of the coronary artery, typically ranging from 20 to 30 mm in length with varying degrees of severity. Finally, type 3 SCAD is characterized by focal or tubular stenosis, resembling the appearance of atherosclerosis in coronary angiography [49]. In cases of both Type 2 and Type 3 SCAD, additional assessment using intravascular imaging methods like optical coherence tomography (OCT) or intravascular ultrasound (IVUS) might be necessary to verify the existence of an intramural hematoma or false lumen, especially when these conditions are not clearly visible on angiography [49]. In the absence of type 1 SCAD, it has been suggested to evaluate type 2 SCAD with intracoronary nitroglycerin, followed by OCT or IVUS if stenosis persists [51].

Among patients with SCAD, the vast majority (90%) are women, with a mean age of approximately 50 years. Notably, conventional cardiovascular risk factors are relatively uncommon in these individuals, a pattern that shares similarities with the broader population of patients at risk for MINOCA [52,53]. Of note, SCAD accounts for nearly 35% of MI occurring in women aged less than 50 years old [54]. Consequently, it should be examined consistently in the differential diagnosis of MINOCA.

SCAD has been associated with various conditions, including factors that make coronary wall structures more susceptible to dissection and stressors that may act as triggers for the dissection. Several risk factors for SCAD recurrence have been identified, such as hypertension, fibromuscular dysplasia, anatomical variations of the coronary arteries, and a history of migraine [55,56,57,58]. The peripartum period is considered a predisposing condition for SCAD due to changes in the intima-media composition of blood vessels, which are associated with the increase in hormonal levels of progesterone. These hormonal changes can weaken the arterial walls, making them more vulnerable to spontaneous dissection during or after childbirth.

### 2.5. Coronary Artery Embolism

Coronary artery embolism (CAE) has been identified as a cause of MINOCA. Single small emboli or multiple microemboli can arise from the lysis of angiographically visible or nonvisible partial non-occlusive thrombi formed on disrupted nonsignificant epicardial plaques. Additionally, direct coronary embolism may stem from a thrombus located in the left atrium, left atrial appendage, or left ventricle, especially in the context of stasis resulting from conditions such as atrial fibrillation, left ventricular dysfunction, or left ventricular noncompaction [3]. Subsequent migration of thrombus to the epicardial coronary arteries can lead to MI. When such embolic events result in the occlusion of a large coronary vessel, it is classified as MI-CAD. On the other hand, occlusion of a small-caliber distal vessel or branch vessel may not be easily visualized on coronary angiography, leading to a clinical diagnosis of MINOCA. Additionally, paradoxical embolization through an atrial septal defect or patent foramen ovale (PFO) can also give rise to MINOCA [11]. Of note, a distal lesion in a single coronary artery may point towards an embolic etiology through the PFO [2,3,59], especially in patients with a high-risk of paradoxical embolism (RoPE) score [60].

Clinicians need to be vigilant about the possibility of coronary artery embolism in patients experiencing a myocardial infarction (MI) and presenting risk factors for thromboembolism. These risk factors include conditions like atrial fibrillation, prosthetic heart valves, atrial septal defect, left-sided valvular diseases (such as infectious endocarditis, aortic or mitral calcification), intracardiac tumors, and various thrombophilic disorders (e.g., factor V Leiden, protein C and protein S deficiency, factor XII deficiency, malignancy, systemic lupus erythematosus, antiphospholipid syndrome) [61,62]. When valvular dysfunction is suspected as the cause of myocardial infarction (MI) or coronary artery embolism, obtaining a transesophageal echocardiogram (TEE) is recommended. TEE provides detailed imaging of the heart structures, including the valves, and can help assess valvular function and the presence of potential embolic sources such as vegetation or tumors [36,61,62].

Lastly, iatrogenic CAE may arise due to clot formation on catheters during prolonged invasive coronary or intracardiac procedures, especially when proper anticoagulation is not maintained and/or catheters are inadequately flushed. Importantly, CAE can also result from nonthrombotic material, including calcifications, fatty emboli, and gas, that can be introduced during invasive procedures [63,64,65,66].

## 3. MINOCA Mimics

Various cardiac conditions, such as takotsubo syndrome, myocarditis, and pulmonary embolism, can lead to chest discomfort and elevated troponin levels, mimicking a clinical presentation of MI. These conditions could be erroneously misclassified as MINOCA. Therefore, it is crucial for clinical providers to proactively pursue diagnostic testing to differentiate and identify these non-MI mimics.

### 3.1. Takotsubo Syndrome

Takotsubo syndrome is a unique and reversible condition characterized by left ventricular dysfunction that can imitate the presentation of acute myocardial infarction (MI). Patients with Takotsubo syndrome may exhibit elevated troponin levels, ischemic changes on the electrocardiogram (ECG), and similar symptoms to those seen in acute MI. The prevalence of TTS is estimated to be approximately 2–3% among patients presenting with suspected ACS [67]. Usually, takotsubo syndrome manifests in postmenopausal women and is preceded by a physical or emotional trigger. It leads to left ventricular wall motion abnormalities that are disproportionate to the peak troponin levels [67]. B-type natriuretic peptide is generally elevated. TTS patients present a typical global medio-distal akinesia with preserved contractility of the basal segments of the left ventricle [67]. The exact underlying mechanisms of takotsubo syndrome remain not fully comprehended, but it is known to differ from MI since it is not linked to atherosclerotic vascular disease. The prevailing explanations point to neurohormonal stunning and/or microvascular spasms as primary factors, with the autonomic nervous system playing a significant role in the condition’s pathophysiology. Additionally, some patients may experience a type of hypertrophic cardiomyopathy characterized by dynamic outflow tract obstruction and increased afterload, potentially contributing to or causing takotsubo syndrome [68]. Thorough examination of coronary angiography is of utmost importance in patients suspected of having takotsubo syndrome. This is crucial because, in a small percentage of these cases, SCAD of the left anterior descending coronary artery has been identified only after a meticulous review conducted by experts [69,70]. Once a diagnosis of takotsubo syndrome has been confirmed, the term MINOCA no longer applies. CMR imaging findings in patients with TTS reveal myocardial edema, which is evident as high signal intensity in T2-weighted images within the same area of the wall motion abnormality. Notably, a classic characteristic of TTS is the absence of late gadolinium enhancement (LGE), and this absence is considered a significant criterion for diagnosing TTS [71].

### 3.2. Myocarditis

Myocarditis is a frequent underlying cause of chest pain and elevated troponin levels in patients provisionally diagnosed with MINOCA. A recent comprehensive systematic review, which encompassed 27 studies and involved 2866 MINOCA patients who underwent CMR, revealed that the prevalence of myocarditis among these individuals was approximately 34.9% (with a 95% confidence interval ranging from 27.8% to 42.4%) [66]. In a patient data meta-analysis of nine studies, it was observed that individuals with angiographically normal coronary arteries had a significantly higher pooled prevalence of myocarditis compared to those with nonobstructive CAD (51% versus 23%, *p* < 0.001). The age- and sex-adjusted odds ratio for this association was 2.30, with a 95% confidence interval ranging from 1.12 to 4.71. Moreover, within the MINOCA patient population, it was found that younger individuals and men were more likely to have myocarditis than women and older patients [66]. In a different study focusing on patients with a provisional diagnosis of MINOCA, it was observed that ST-elevation myocardial infarction (STEMI) at the time of presentation did not show an association with a higher prevalence of myocarditis as assessed by CMR. In other words, the presence of STEMI did not seem to significantly impact the likelihood of concurrent myocarditis in these patients [72]. A final diagnosis of myocarditis takes precedence over the provisional pre-CMRI MINOCA designation. When myocarditis is confirmed through CMRI, it becomes the primary diagnosis, and the initial provisional MINOCA diagnosis is replaced. However, it is important to note that in some cases of CMRI-confirmed myocarditis, coronary artery spasm may still be observed.

### 3.3. Pulmonary Embolism

Pulmonary embolism (PE) should be considered as a potential diagnosis in all patients with a provisional diagnosis of MINOCA. If patients exhibit unexplained tachycardia, tachypnea, or hypoxia, it is essential to perform D-dimer measurement or computed tomography pulmonary angiography to assess for the possibility of PE, either before or after coronary angiography. Even in the absence of these specific signs, the potential diagnosis of PE should still be considered in the context of MINOCA.

However, it is worth noting that in a series of 100 patients with MINOCA who underwent routine computed tomography pulmonary angiography, none of them were found to have PE. This underscores the importance of careful evaluation and appropriate testing in patients presenting with suspected MINOCA to ensure that life-threatening conditions like PE are not overlooked [73].

### 3.4. Thrombophilia

Hereditary thrombophilia appears to be a possible contributing factor to the pathogenesis of MINOCA (myocardial infarction with non-obstructive coronary arteries). A pooled analysis of eight studies that investigated screening for inherited thrombophilia in MINOCA patients found that approximately 14% of the patients had an inherited thrombotic disorder.

The breakdown of specific thrombotic disorders identified in these patients was as follows:

- Factor V Leiden: present in 12% of the cases;
- Protein C or S deficiency: identified in 3% of the patients;
- Factor XII deficiency: observed in 3% of the cases [36].

Absolutely, acquired thrombophilia can also contribute to the development of MINOCA. A study that conducted a comprehensive assessment for thrombophilia in 84 MINOCA patients more than three months after their heart attack revealed the following results:

- Antiphospholipid syndrome: identified in 15.5% of the patients;
- Inherited thrombophilia: found in 23.8% of the cases [74].

These findings indicate that acquired thrombotic disorders, including antiphospholipid syndrome and inherited thrombophilia, both play a role in a significant proportion of MINOCA patients. The study highlights the importance of considering and investigating various thrombophilic factors in the evaluation of MINOCA to better understand its underlying pathogenesis and optimize patient management.

### 3.5. Oxygen Supply/Demand Imbalance Myocardial Infarction

In certain patients, ischemic myocardial necrosis can occur even in the absence of obstructive CAD. This happens due to an imbalance between the supply of oxygen to the heart muscle and its demand, without any specific evidence of mechanisms causing a significant reduction in coronary blood flow. This type of acute MI is classified as type 2 MI and is typically a result of systemic conditions that lead to an increase in myocardial oxygen demand and/or impaired oxygen delivery. Examples of such conditions include sepsis, tachyarrhythmias, anemia, hypotension, and hypoxia. This type of MI usually occurs in the presence of CMD or epicardial vasoconstriction.

Patients with type 2 MI are typically older, with a higher proportion of women, and they often have more comorbidities compared to patients with AMI caused by atherothrombotic disease, where there is a blockage in the coronary arteries due to atherosclerosis and blood clot formation. Type 2 MI represents a distinct category of heart attack with different underlying causes and patient characteristics [75,76]. The approach to managing type 2 MINOCA involves addressing the underlying conditions and eliminating the triggers, with decisions about long-term therapy made following a comprehensive evaluation of the cardiac status during the acute phase.

## 4. Diagnostic Tools in MINOCA

Establishing a diagnosis of MINOCA can be challenging since traditional MI diagnostic approaches rely on evidence of significant coronary artery obstruction; hence, the optimization of MINOCA diagnostic criteria is still under investigation. Due to the inherent heterogeneity of MINOCA as a disease entity, the extent of the diagnostic strategies implemented often varies depending on local non-standardized practices [76]. Hence, the current ESC guidelines on the management of non-ST segment elevation acute coronary syndromes include a dedicated section on MINOCA with a recommended diagnostic algorithm (class of recommendation: I). MINOCA is briefly defined as MI with (1) elevated cardiac biomarkers (typically cardiac troponin > 99th percentile of the upper reference level with a rise or fall in the level on serial assessment), (2) non-obstructive coronary arteries as per angiographic guidelines, and (3) no alternate diagnosis for the acute clinical presentation [20]. Table 1 provides the expanded criteria for the MINOCA definition, as stated in the latest ESC guidelines [20].

Current guidelines encourage clinicians to follow a diagnostic stepwise algorithm to differentiate true MINOCA from alternative diagnoses in all patients with an initial working diagnosis of MINOCA (class of recommendation: I) [20]. To that end, several diagnostic stepwise approaches have been developed by consensus of expert opinions, having as a common ground that the initial MINOCA diagnosis should be confirmed or ruled out based on the results of subsequent investigations [12,77,78,79,80,81]. More specifically, after establishing the working diagnosis of MINOCA through an invasive coronary angiogram, the diagnostic work-up should rapidly begin with the exclusion of potential alternative diagnoses within a clinical context (e.g., sepsis, critical illness, toxicity, stroke, pulmonary embolism, cardiac contusion, aortic dissection, heart failure, non-cardiac troponin rise, heterophilic antibody-associated troponin rise). After excluding those cases, a review of the angiographic findings and functional assessment of the left ventricle (LV) with echocardiogram or cardiac ventriculography could be the next step. Then multimodality imaging (CMR and IVUS/OCT with or without intracoronary functional testing) could help clinicians identify specific ischemic diagnoses or non-ischemic mimics. The preference for invasive (mostly OCT) versus non-invasive imaging (CMR) is based on the local availability/expertise and the clinical/angiographic presentation of each patient. More detailed information for each diagnostic step is presented in the following subsections.

### 4.1. Angiographic Re-Appraisal, Clinical Evaluation and Laboratory Testing

As noted above, after the establishment of a MINOCA working diagnosis, the coronary angiograms should be re-evaluated for missed significant stenosis or side branch occlusion, while life-threatening conditions of acute myocardial injury that could mimic an AMI should be rapidly and systematically excluded in a multidisciplinary clinical approach [3]. Additional D-dimer and thrombophilia testing (with or without CT pulmonary angiography), erythrocyte sedimentation velocity, C-reactive protein, and natriuretic peptide testing along with monitoring trends in cardiac troponin could be considered for evaluation where appropriate [20]. The presence of heterophilic antibodies as a cause of false positive troponin rise might also be considered in laboratories with availability for the use of blocking agents to control those antibodies [82]. In the future, “omics” approaches (i.e., genomics, epigenomics, transcriptomics, proteomics, metabolomics, lipidomics) might provide further insights into the diagnosis of MINOCA. However, significant research efforts coupled with artificial intelligence tools are still needed to assess “omics” applicability in such a small population subset [83].

### 4.2. Echocardiography

Transthoracic echocardiogram could provide direct and indirect information (e.g., dilated ventricles, atrial size, increased wall thickness, LV wall motion, impaired systolic function, and pericardial effusion), which may help the clinician exclude non-ischemic causes (e.g., Takotsubo syndrome and myocarditis) and indicate potential causes responsible for the acute presentation. Also, regional wall motion abnormalities may indicate an epicardial cause of MINOCA or other specific causes not belonging to the MINOCA definition. Additionally, if coronary artery embolism is clinically suspected, then a transesophageal echocardiogram can be used to identify any intracardiac clot or valvular vegetation [84].

### 4.3. Cardiac Ventriculography

In patients with suspected MINOCA, the performance of cardiac ventriculography aims to rule out Takotsubo syndrome (stress cardiomyopathy) as an alternative diagnosis [85]. Hence, the decision to perform cardiac ventriculography (at the price of more contrast medium administration) depends on the clinical likelihood of presenting with Takotsubo syndrome [86].

### 4.4. Intracoronary Imaging

According to the current ESC guidelines, intracoronary imaging using IVUS or OCT can be valuable in identifying potential causes during coronary angiography, particularly in cases where thrombus, plaque rupture, erosion, and SCAD are suspected [20]. Nevertheless, in real-world scenarios, OCT appears to offer higher resolution than IVUS and may be more beneficial in identifying culprit lesions based on suggestive signs such as SCAD, rupture, erosion, erupted nodules, cavities, layered plaque, and residual thrombus) [87,88]. However, the selection of vessels to image presents a challenge, as reliably identifying the culprit vessel based on electrocardiographic or angiographic findings is difficult in several MINOCA cases. Consequently, a three-vessel intracoronary investigation might be necessary to achieve the most comprehensive diagnostic results [77].

In a recent landmark study by Reynolds et al., almost half of the MINOCA patients remained with an undefined diagnosis based on OCT findings [23]. Probably, intracoronary imaging has the highest sensitivity when performed early in the course of MINOCA (maybe during the first angiogram) or offers particular diagnostic benefits for only some cases [84]. Specifically, patients who exhibit at least a mid-range stenosis (e.g., 10% to 50%) in any of the major epicardial vessels are considered the most suitable candidates for OCT imaging. This is to ensure that any plaque-induced events have not gone unnoticed. Additionally, OCT allows for the detection of coronary spasms through evidence of intimal bumping. When the findings from OCT are inconclusive, consideration may be given to conducting invasive tests to evaluate microvascular coronary disease or coronary vasospasm, depending on local availability and expertise in performing such procedures [80].

### 4.5. Invasive Functional Coronary Tests

Based on the current ESC guidelines, intracoronary acetylcholine or ergonovine provocative testing may be performed when the coronary or microvascular spasm is suspected [9,77,89]. Provocative testing enables the evaluation of coronary spasm and may be considered at the time of diagnostic angiography for MINOCA or during a following invasive evaluation. The combined use of OCT may also correlate vasospastic angina with concurrent non-obstructive atherosclerotic plaques [9].

Moreover, the assessment of coronary physiology through invasive [coronary flow reserve (CFR), index of microvascular resistance (IMR), and absolute coronary blood flow] and non-invasive methods [positron emission tomography (PET), CMR, and Doppler echocardiography] may identify microvascular dysfunction responsible for myocardial ischemia [78,81]. The role of fractional flow reserve (FFR) or instantaneous wave-free ratio (iFR) measurement has not yet been studied systemically in patients with MINOCA.

### 4.6. Cardiac Magnetic Resonance

In the current ESC guidelines, it is recommended to perform CMR in all MINOCA patients without an obvious underlying cause (class of recommendation: I) [20]. CMR is characterized as one of the key diagnostic tools and the gold standard among non-invasive diagnostic tools in the evaluation of suspected MINOCA, permitting the differential diagnosis of Takotsubo syndrome, myocarditis, or true AMI [90,91,92].

More specifically, CMR, a non-ionizing radiation test, can assess myocardial perfusion, ventricular function, and the underlying mechanism of myocardial injury [either reversible (e.g., inflammation, edema) or irreversible (e.g., necrosis, fibrosis)]. CMR interpretation can also differentiate between ischemic patterns (following vascular territory and appearing as edema or fibrosis affecting the subendocardial or transmural myocardium) and non-ischemic patterns (not following vascular territories and appearing as subepicardial or intra-myocardial edema or fibrosis) via T1-, T2-, extracellular volume- (ECV) sequences and (free-breathing) late gadolinium enhancement (LGE) techniques [3,12]. In the near future, CMR with quantitative parametric mapping, including the promise of CMR fingerprinting, might also have a role to play in MINOCA diagnostics [93].

Studies investigating the diagnostic discrimination in MINOCA have highlighted the critical importance of timely CMR investigation, ideally during the index hospitalization or within the first two weeks from the acute presentation, to avoid false negative results (e.g., missed findings of myocardial edema or myocarditis) [23,77,78]. Real-world evidence suggests that CMR can identify an underlying etiology in almost 75% of patients presenting with MINOCA [23]; thus, CMR should be offered early in the diagnostic pathway, particularly in centers with CMR availability and expertise [77].

Aiming to reach optimal diagnostic yield in suspected cases with MINOCA, intracoronary imaging (mostly OCT) could be subsequently offered for further clarification of the underlying mechanism [77]. A normal early CMR may prompt an invasive approach, including intracoronary imaging and/or functional (provocative) tests. The combination of OCT and CMR could result in the identification of MINOCA etiology in up to 85% of the total population [23]. Coupling CMR with intracoronary imaging seems to hold promise since some MINOCA patients have smaller infarct sizes, which might be too small to visualize on CMR [77]. Moreover, in cases with inconclusive findings by early performed intracoronary imaging, CMR could also unravel the final diagnosis in a great proportion of cases [23].

### 4.7. Coronary Computed Tomography Angiography

Coronary computed tomography angiography (CCTA) has become a key imaging modality in patients with stable chest pain due to its high diagnostic yield [94,95]. Currently, there is no evidence arguing for a systematic use of (CCTA) in MINOCA. Its current utility seems to be limited to aiding in the decision of whether to prescribe statin therapy to a MINOCA patient with angiographically normal coronary arteries [77]. However, more recently, CCTA utilization in MINOCA gained interest due to its potential for recognizing high-risk atherosclerotic plaques invisible at ICA and assessing perfusion data, inflammation, and peri-coronary fat tissue [77,84,96,97]. CCTA also excels in monitoring plaque progression, regression, or stabilization, with results comparable to IVUS [98]. The additional ability of CCTA for a holistic evaluation of the entire coronary vessel for atherosclerosis, rather than a segmental approach by IVUS or OCT, could be crucial to identify and predicting ischemia [99,100]. Additionally, the characteristics of the coronary artery detected by CCTA, such as plaque volume and distribution, maximal luminal stenosis, as well as pericoronary inflammation, could provide useful information beyond CMR [97,101,102]. Of course, in cases where cardiac catheterization, CMR, and intracoronary imaging are unavailable or contraindicated, CCTA might play a predominant role by effectively ruling out obstructive CAD [103,104,105]. Nonetheless, the outcomes of the ongoing MINOCA-GR trial might hopefully shed light on the utility of CCTA in MINOCA patients [101].

The optimal diagnostic tool linked with maximum diagnostic yield in each specific cause of MINOCA is summarized in Table 2.

## 5. Cause-Targeted Treatment

### 5.1. The General Approach to Management in Patients with MINOCA

**Figure 1:**
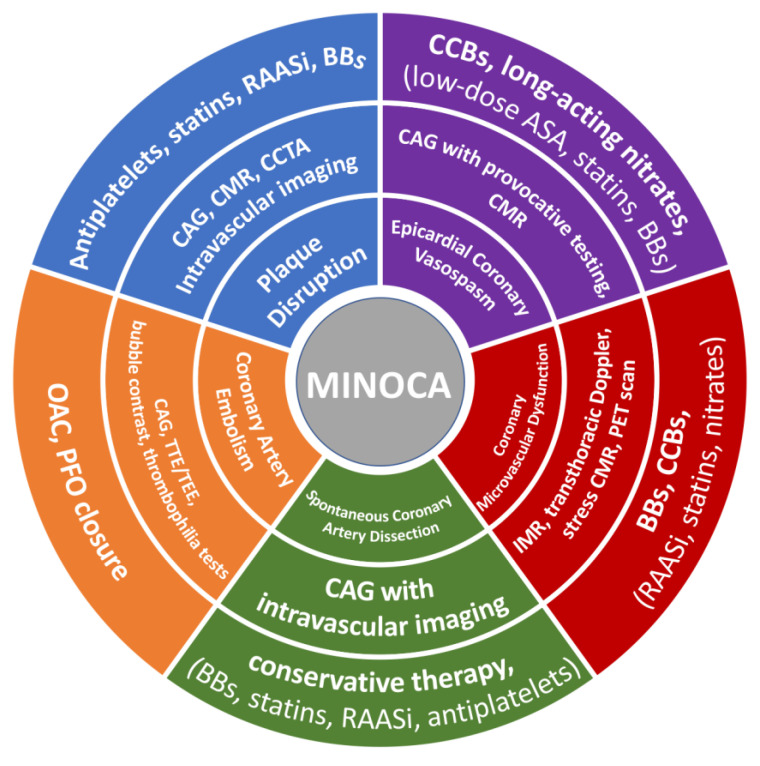
Diagnostic tools and therapeutic strategy based on the etiology of MINOCA. CAG—coronary angiogram; CMR—cardiac magnetic resonance; CCTA—coronary computed tomography angiography; ASA—acetylsalicylic acid; BBs—beta-blockers; RAASi—renin–angiotensin adrenergic antagonists; CCBs—calcium channel blockers; OAC—oral anticoagulation; PFO—patent foramen ovale; TTE—transthoracic echocardiography; TEE—transesophageal echocardiography; PET—positron emission tomography; IMR—index of microvascular resistance.

The secondary prevention of MI is based largely on clinical practice guidelines that were derived from studies analyzing patients with MI and obstructive CAD [106]. Currently, there are no RCTs dedicated to MINOCA. Therefore, there is a lack of knowledge regarding secondary preventive therapies in patients with MINOCA. Consequently, pharmacological therapy for secondary prevention of MI is administered less often at discharge in patients with MINOCA compared with their MI-CAD counterparts, while a significant heterogeneity in the management of these patients is observed in clinical practice [107,108]. It is important to note that current expert recommendations are not aligned with each other, and in some cases emerge, conflicting opinions and differences in interpretations. Of note, ESC guidelines for the management of non-ST segment elevation acute coronary syndromes recommend the use of conventional secondary prevention medications for MINOCA, similar to those with MI-CAD, when the specific underlying cause has not yet been identified [20]. Moreover, ESC guidelines recommend routine use of aspirin, statins, and CCB for vasospasm [3], while AHA suggests that statins and antiplatelet use should only be reserved for MINOCA caused by plaque disruption while it should be avoided in type 2 MI, as it might be contraindicated [109]. At present, targeted therapies in MINOCA based on the underlying pathophysiologic mechanism have not been adequately analyzed yet. Figure 1 illustrates the available diagnostic tools and optimal therapeutic treatment based on the etiology of MINOCA.

### 5.2. Plaque Disruption

Recommendations suggest that patients presenting with MINOCA due to coronary plaque disruption should be treated with cardioprotective agents as per acute MI guidelines, similarly to patients who suffered acute coronary syndrome [106,110]. This includes DAPT, beta-blockers, statin, and RAAS inhibitors (in the presence of impaired left ventricular function) [111]. Treatment with DAPT in MINOCA is based on RCTs on acute MI, which showed the benefit of adding a P2Y12 receptor inhibitor to aspirin without requiring identification of obstructive CAD [112,113]. Even though plaque disruption is common in MINOCA, a retrospective cohort study of MINOCA patients registered in the SWEDEHEART database showed that the use of DAPT did not provide any prognostic benefit [112,113,114]. Nevertheless, it is important to note that this result was obtained from the entire MINOCA cohort, whereas DAPT might be beneficial specifically for patients with a thromboembolic cause of MINOCA.

A pilot study by Prati et al. compared the effectiveness of DAPT versus angioplasty and stenting in 31 patients with OCT-detected culprit plaque erosion. The study demonstrated a low rate of adverse events and revascularization in patients treated with DAPT alone [115]. The EROSION study further confirmed these findings, showing that DAPT with aspirin and ticagrelor significantly reduced thrombus volume and resulted in a low rate of adverse events at 30 days. Moreover, at the 1-year follow-up, 92.5% of patients with AMI caused by plaque erosion and managed with DAPT without stenting remained free of MACE. However, it is worth noting that these studies included both obstructive and nonobstructive lesions without specifying the exact number of nonsignificant lesions (stenosis < 50%), and they relied on surrogate primary endpoints. Therefore, to validate these results, dedicated RCTs in MINOCA patients with sufficient sample size and power for clinical outcomes are necessary [116,117]. A recent meta-analysis of five observation studies in MINOCA demonstrated a neutral prognostic effect of DAPT [118]. Another meta-analysis aggregated data from two studies to assess the impact of DAPT on the outcomes of MINOCA patients over a median follow-up of 24 months [119,120,121], indicating a significant reduction in all-cause death in MINOCA patients [hazard ratio (HR) 0.73, 95% confidence interval (CI) 0.55–0.98]. However, this meta-analysis should be approached with caution as it only evaluated two studies, with one study carrying much more weight than the other (98.6% vs. 1.4%), which could skew the results. Additionally, in one of the studies, only CMR was performed to differentiate between ischemic patterns of myocardial damage and alternative conditions like Takotsubo syndrome or myocarditis. In summary, the current evidence does not support the routine administration of DAPT in all MINOCA patients [3,77]. The lack of effectiveness of DAPT in MINOCA patients is a paradox that requires further investigation and exploration through randomized trials, specifically focusing on MINOCA caused by transient thrombosis on disrupted, nonsignificant coronary plaques.

The use of beta-blockers in conjunction with DAPT may prove to be beneficial for patients with MINOCA caused by thromboembolic mechanisms, similar to the approach for the entire population of AMI. It is widely recognized that AMI patients exhibit increased sympathetic activation, which can significantly contribute to cardiovascular events [122], and beta-blockers may improve clinical outcomes by countering the negative effects of the sympathetic nervous system on the infarcted and ischemic myocardium, primarily by reducing myocardial oxygen demands and increasing myocardial resistance to ischemic injury. However, current recommendations for using beta-blockers in the entire population of AMI patients are not consistent. American guidelines suggest routine treatment with beta-blockers, while European guidelines limit the recommendation to patients with heart failure or left ventricular systolic dysfunction [20,110,111]. In the case of MINOCA, it should be noted that no randomized controlled trial has yet evaluated the effect of beta-blockers treatment in this specific setting [110,123]. Nonetheless, a study by Lindahl et al. found that beta-blocker treatment in MINOCA patients was associated with a 14% reduction in MACE, even if statistical significance was not reached [120]. Moreover, data from a multicenter national registry demonstrated that the use of beta-blockers was associated with a low frequency of MACE during a median follow-up of 8.5 years in MINOCA patients [124]. In a meta-analysis by Samaras et al., beta-blockers were not associated with a lower risk of all-cause mortality or MACE [118].

RAAS inhibitors are another class of drugs that are commonly administered post-AMI, with angiotensin-converting enzyme inhibitors being particularly effective in improving survival according to several randomized controlled trials of AMI patients [10,125]. Although several observational studies have indicated the beneficial effect of RAAS inhibitors in MINOCA, certain limitations should be taken into consideration, including variability in the definition of MINOCA across studies and the inclusion of patients with non-ischemic causes of troponin elevation [20,111,114].

Finally, statin therapy is recommended for patients with MINOCA as a result of thromboembolic complications [5]. The benefit of statins in the entire AMI population is well recognized. The lack of randomized controlled trials specifically focused on MINOCA patients yielded conflicting results from the existing observational studies [10,120,121,123,126]. However, a recent meta-analysis demonstrated a reduction in mortality risk associated with statin use in MINOCA [117]. In the SWEDEHEART registry, statins reduced various cardiovascular endpoints, including all-cause mortality [120]. In the context of atherothrombotic MINOCA, statins are believed to stabilize unstable plaques enriched with lipid content. This effect is achieved through lipid reduction (the primary goal of statins) and various pleiotropic mechanisms, including anti-inflammatory, antioxidant, and antithrombotic effects [127]. For patients with atherothrombotic MINOCA experiencing statin intolerance or insufficient cholesterol control, the use of ezetimibe and PCSK-9 inhibitors should also be considered [22,106,110,128].

### 5.3. Epicardial Coronary Vasospasm 

The management of CAS involves implementing certain lifestyle changes to eliminate conditions that may promote CAS induction. These changes primarily focus on smoking cessation and reducing alcohol consumption consistently. Additionally, it is important to avoid the use of certain substances and drugs, such as cocaine, sympathomimetic agents, beta-blockers, parasympathomimetic agents, ergot alkaloids, and the chemotherapeutic drug 5-fluoro-uracil, as they may trigger or exacerbate CAS [129,130].

CCBs are the most effective therapeutic option in coronary spasms not only due to their antianginal properties but also because they have been associated with improved cardiovascular outcomes in patients with vasospastic angina [131,132]. CCBs typically alleviate angina symptoms and lead to improved clinical outcomes in patients with CAS. Therefore, discontinuing an effective CCB therapy can have adverse consequences for these patients. A recent study focusing on MINOCA patients revealed that two-thirds of all deaths and 60% of cardiac deaths occurred in patients who initially received CCBs and had a positive provocative test for CAS but later reduced or stopped their CCB treatment during the follow-up period [9].

The benefit of nitrates in alleviating spasms during the acute phase has been well established, while their long-term effects remain unclear, possibly due to tolerance [133]. To address this issue, it is recommended nitrates be administered in an asymmetrical manner throughout the day when used in combination with CCBs. This approach aims to cover the period when angina attacks are most likely to occur, providing relief during these times. However, in order to ensure optimal results, it is essential to create a nitrate-free period in the treatment schedule to allow for the restoration of the vascular sensitivity to their dilating effect.

Low-dose aspirin is effective in treating coronary vasospasm by inhibiting thromboxane-A2-mediated vasoconstriction. However, caution should be exercised with large doses of aspirin, as they may exacerbate vasospasms through prostacyclin inhibition [134,135]. Additionally, the addition of statins, cilostazol, and nicorandil (an ATP-sensitive potassium channel modulator with nitrate-like properties) may offer potential benefits for patients with vasospasm [136]. Specifically, several studies have indicated that adding statins to vasodilator therapy may enhance symptom control and improve outcomes in patients with CAS. This benefit is attributed to the pleiotropic effects of statins, particularly the improvement in endothelial function resulting from decreased oxidative stress and inflammation [121,137]. However, it is important to note that no RCTs have been conducted specifically on the beneficial effect of statins in patients with vasospastic angina. Therefore, at present, the use of statins in MINOCA caused by CAS cannot be recommended unless they are indicated for other medical reasons. 

### 5.4. Coronary Microvascular Dysfunction

The evidence necessary to appropriately guide the management of MINOCA due to CMD is scarce. Clinical trials dealing with this heterogeneous disorder have included patients with ischemia with non-obstructive CAD rather than MINOCA [136]. Even in the absence of randomized controlled trials, b-blockers appear to be the most effective long-term prevention therapy in CMD, improving angina and exercise tolerance [138]. CCBs have also been found to provide optimal symptomatic treatment in these patients [139]. Conventional vasodilator drugs, such as nitrates, are less effective on the microvasculature associated with coronary microvascular dysfunction [136]. However, the aforementioned therapies are limited to CMD patients with stable angina [41,140,141]. Several unconventional antianginal therapies have shown their beneficial effect by improving endothelial function (e.g., l-arginine) or promoting microvascular vasodilation (e.g., ranolazine) [141,142]. Specifically, ranolazine use appeared to be safe, reduced angina, and improved exercise performance significantly in patients with microvascular angina [142]. Furthermore, the available evidence supporting the use of angiotensin-converting enzyme inhibitors (ACEIs) or angiotensin receptor blockers (ARBs) as effective monotherapy for MINOCA due to CMD is limited. However, several studies have suggested that the combined use of an aldosterone antagonist along with an ACEI or ARB may provide additional clinical benefits in managing CMD [143].

### 5.5. Spontaneous Coronary Artery Dissection

Currently, conservative therapy is preferred in patients with SCAD, even though management is based on expert recommendations due to a lack of RCTs [49,144]. Observational studies have reported that 70% of SCAD resolves on repeat angiography, suggesting that conservative medical treatment and inpatient monitoring are sufficient in most cases [51,145,146]. Coronary revascularization may be considered necessary only in specific cases, such as when there are occluding lesions, the presence of high-risk anatomical features (involvement of severe proximal sites in the left main coronary artery or proximal left anterior descending artery), low-grade thrombolysis in myocardial infarction (TIMI) flow, ongoing myocardial ischemia with hemodynamic instability, or disease that is unresponsive to medical treatment [147]. Indeed, stent implantation in SCAD cases has been linked to a higher risk of complications, as it may exacerbate the propagation of vessel dissection. In situations where percutaneous coronary intervention (PCI) is deemed necessary, a cut balloon dilation, with or without stent placement, may be considered a more prudent approach [148]. Empiric DAPT could be used for prevention, as intimal tears can be prothrombotic [48]. However, the use of DAPT remains controversial because it could theoretically lead to increased bleeding and propagation of the dissection. B-blockers could be beneficial in SCAD by minimizing arterial wall stress, which may reduce adverse events and SCAD recurrence [149]. Other therapies with potential yet not proven beneficial effects in SCAD could be RAAS inhibitors in patients with impaired left ventricular function and statins in the presence of dyslipidemia [50].

### 5.6. Coronary Artery Embolism

The management of CAE requires a personalized approach, taking into consideration various factors, such as patient-specific characteristics, the timing of presentation, and the presence or absence of other embolic sources. Currently, there are no prospective, randomized controlled trials that specifically advocate for long-term anticoagulation or antiplatelet therapies as standard treatment for MINOCA caused by coronary artery embolism. However, it is recommended that patients with MINOCA attributed to CAE receive anticoagulant therapy, likely with one of the oral anticoagulant drugs [150]. In cases of CAE, anticoagulation should be continued for a duration of 3 months if there are no ongoing procoagulant factors or persistent risk factors. However, if persistent risk factors for CAE are present, consideration should be given to long-term oral anticoagulation therapy. In situations where paradoxical coronary embolism (PCE) is suspected due to a PFO, percutaneous closure of the PFO can be performed as a treatment approach. Finally, anticoagulation therapy may be appropriate for the prevention of embolic events in left-side origin coronary embolism or for long-term pharmacological treatment [129].

## 6. Prognosis in MINOCA

There is increasing evidence that the prognosis of patients with MINOCA is alarming, unlike the common perception that non-obstructive coronary artery disease theoretically carries a good prognosis [36]. Recent meta-analyses of MINOCA studies demonstrated pooled 12- and 25-month all-cause mortality rates of 3.4% and 2.0%, respectively [151,152]. Similar findings of 2% yearly mortality rates were reported in two large multicenter observational studies in MINOCA [153], while PURSUIT reported a 2.2% rate of mortality or recurrent myocardial infarction at six months [154]. Data from the SWEDEHEART registry revealed a 4.5-year mortality of 14% in MINOCA [155]. Unfavorable outcomes in MINOCA may be due to the suboptimal inclusion of MINOCA-mimicking conditions, particularly myocarditis and Takotsubo cardiomyopathy, in these observational studies. Nevertheless, high mortality rates may also be explained by the potential presence of unstable plaque ruptures, a high-risk epicardial MINOCA subset, which bears a worse prognosis than a fibrous cap presence [156]. Epicardial vasospasm and abnormal acetylcholine provocation testing have also been associated with unfavorable outcomes in MINOCA [9].

### 6.1. MINOCA vs. Other Populations (MI-CAD, Angina, General Population)

Comparing the prognosis between patients with MINOCA and patients with MI and obstructive CAD could be particularly challenging due to the different underlying pathophysiological mechanisms. As previously discussed, MINOCA is a highly heterogeneous condition in which, in contrast to MI with obstructive CAD, the thrombo-atherosclerotic mechanism does not dominate [23]. Most studies have demonstrated that patients with MINOCA have better short- and long-term outcomes than patients with MI and significant CAD [19,152]. A recent meta-analysis reported that patients with MI and obstructive CAD have more than 2-fold higher annual mortality rates than their MINOCA counterparts [151]. Nevertheless, these findings are not consistent among all reports. In the VIRGO study, mortality rates were comparable between MINOCA and MI-CAD patients [157]. A large observational study showed that MINOCA patients had a similar prognosis as MI-CAD patients with one- or two-vessel angiographic disease [158]. A propensity-matched analysis reported that MINOCA patients had a higher rate of 12-month all-cause mortality, especially non-cardiac deaths, compared with patients with NSTEMI and CAD [159]. Furthermore, it has been reported that almost 1 out of 4 patients suffering from MINOCA will experience angina at 12 months at similar rates to patients with MI-CAD [160]. In any case, the rate of long-term cardiovascular adverse events is concerning, given that patients with MINOCA tend to be younger and healthier than patients with MI-CAD [36,120]. Compared with the annual mortality of 0.3% of patients with chronic angina and angiographically non-obstructed coronary arteries, patients with MINOCA have considerably higher mortality rates [161]. Patients with MINOCA are also at higher risk of short- and long-term mortality and risk of recurrent events than the general population [162,163].

### 6.2. Underutilization of Secondary Prevention Medication in MINOCA

It has been reported that long-term use of conventional secondary prevention medications was lower in patients with MINOCA than in patients with MI-CAD [164,165]. Another study reported that the use of secondary preventive medication in post-MI patients was significantly lower in non-obstructive CAD than in obstructive CAD [166]; the latter study did not report an intergroup difference in CCB use, which may disclose that treatment in MINOCA patients may be focused more on prevention of recurrent vasospasm and angina [166]. A report from the COAPT study confirmed the lower use of secondary prevention therapies in MINOCA, especially in the subgroup with angiographically normal coronary arteries [167].

## 7. Future Implications

Pharmacological treatment in patients with MINOCA can vary widely, given the absence of randomized clinical trials. Guidelines for secondary prevention therapies in patients with MINOCA are warranted to guide routine clinical practice. The impact of b-blockers and renin–angiotensin system modulating agents on MACE in patients with suspected MINOCA is currently being assessed in the MINOCA-BAT randomized, parallel, open-label, multicenter trial (NCT03686696). Furthermore, the StratMed-MINOCA trial aims to enroll 150 patients diagnosed with MINOCA and who show evidence of coronary microvascular disease. These patients will be randomly assigned to receive either eplerenone or usual care. The study’s primary objective is to assess the change in N-terminal pro-brain natriuretic peptide within each patient at 30 days and six months. The evaluation of MACE will be considered a secondary endpoint in this trial. 

On the other hand, the PROMISE trial will recruit 180 patients diagnosed with MINOCA and randomly divide them into two groups. One group will receive a precision medicine approach involving coronary optical coherence tomography (OCT), cardiac magnetic resonance imaging (CMRI), and coronary spasm testing. This personalized approach will be used to guide tailored medical therapy. The other group will follow a standard approach to acute coronary syndrome management. The role of CMRI was also demonstrated in a recent large meta-analysis of 26 studies [168], showing an important diagnostic and prognostic value of this imaging modality in patients with MINOCA, proving to be crucial for their diagnosis. Therefore, our review provides valuable insights in light of the emergence of such new studies.

Other novel concepts, such as the role of adaptive immune cells in MI through an accelerated regeneration of injured heart muscle, could have beneficial effects in patients with MINOCA and warrant further investigation [169,170,171,172]. Moreover, investigating each MINOCA subcategory on its own might reveal further patient subgroups of prognostic relevance. Specifically, the hypothesized underlying mechanisms for CMD appear to be heterogenous, including enhanced coronary vaso-constrictive reactivity at the microvascular level, impaired endothelium-dependent, independent coronary vasodilator capacities, and increased coronary microvascular resistance secondary to structural factors. Hence, introducing the concept of morphological versus functional CMD is an intriguing idea, the clinical relevance of which remains to be assessed in future studies.

## 8. Conclusions

MINOCA occurs in almost 1 out of 12 patients who are referred for coronary angiography following AMI. Its pathogenesis is highly variable, beyond the straightforward etiologic mechanism of MI-CAD. Atherosclerosis, thrombosis, coronary artery spasm, and coronary microvascular dysfunction are the major mechanisms of MINOCA. Identifying the underlying mechanism of infarction in MINOCA through multimodality imaging techniques could provide individualized patient management. Optimal secondary prevention medication following MINOCA is uncertain since current guidelines are largely based on data from patients with AMI and obstructive CAD. Clinical trials are warranted to define the optimal treatment of patients with MINOCA.

## Figures and Tables

**Table 1:** Current diagnostic criteria for MINOCA as stated in the current ESC guidelines on management of non-ST segment elevation acute coronary syndromes.

1. AMI (Modified from the ‘Fourth Universal Definition of Myocardial Infarction’ Criteria):	2. Non-Obstructive Coronary Arteries on Angiography [Absence of Obstructive Disease on Angiography (i.e., No Coronary Artery Stenosis ≥ 50%) in Any Major Epicardial Vessel]:	3. No Specific Alternate Diagnosis for the Clinical Presentation:
Detection of a rise or fall in cardiac troponin with at least one value above the 99th percentile upper reference limit AND	Normal coronary arteries (no angiographic stenosis) Mild luminal irregularities (angiographic stenosis <30% stenoses) Moderate coronary atherosclerotic lesions (stenoses >30% but <50%)	Non-ischaemic causes such as sepsis, heart failure, cardiomyopathy, pulmonary embolism, cardiac contusion, aortic dissection, Takotsubo syndrome and myocarditis
Corroborative clinical evidence of infarction as shown by at least one of the following: a. symptoms of myocardial ischemia; b. new ischaemic electrocardiographic changes; c. development of pathological Q waves; d. imaging evidence of new loss of viable myocardium or new regional wall motion abnormality in a pattern consistent with an ischaemic cause; e. identification of a coronary thrombus by angiography or autopsy.		

**Table 2:** Optimal diagnostic tool for MINOCA.

MINOCA Specific Cause:	Helpful Diagnostic Tool:
Atherosclerotic plaque disruption	Invasive Coronary Angiography + Cardiac Magnetic Resonance
Coronary embolism	Invasive Coronary Angiography
SCAD	Invasive Coronary Angiography + intravascular imaging
Epicardial or microvascular spasm	Invasive Coronary Angiography + provocative testing
Coronary microvascular dysfunction	Invasive Coronary Angiography + index of microvascular resistance

SCAD—spontaneous coronary artery dissection.

## Data Availability

No new data were created or analyzed in this study. Data sharing is not applicable to this article.
